# Arteriovenous Length Ratio: A Novel Method for Evaluating Retinal Vasculature Morphology and Its Diagnostic Potential in Eye-Related Diseases

**DOI:** 10.3390/jimaging9110253

**Published:** 2023-11-20

**Authors:** Sufian A. Badawi, Maen Takruri, Mohammad Al-Hattab, Ghaleb Aldoboni, Djamel Guessoum, Isam ElBadawi, Mohamed Aichouni, Imran Ali Chaudhry, Nasrullah Mahar, Ajay Kamath Nileshwar

**Affiliations:** 1 Department of Computer Science, Faculty of Information Technology, Applied Science Private University, P.O. Box 541350, Amman 11937, Jordan; 2 Center of Information, Communication and Networking Education and Innovation (ICONET), American University of Ras Al Khaimah, Ras Al Khaimah 72603, United Arab Emirates; maen.takruri@aurak.ac.ae (M.T.); ghaleb.aldoboni@aurak.ac.ae (G.A.); djamel.guessoum.1@ens.etsmtl.ca (D.G.); 3 College of Engineering, Al Ain University, Al Ain 64141, United Arab Emirates; mohammad.alhattab@aau.ac.ae; 4 Electrical Engineering Department, Ecole de Technologie Superieure, Montreal, QC H3C 1K3, Canada; 5 Industrial Engineering Department, College of Engineering, University of Ha’il, Ha’il 81481, Saudi Arabia; i.elbadawi@uoh.edu.sa (I.E.); m.aichouni@uoh.edu.sa (M.A.); i.chaudhry@uoh.edu.sa (I.A.C.); 6 Computer Science Department, Bahauddin Zakariya University, Multan 60800, Pakistan; nasr@wizinsights.com; 7 Department of Ophthalmology, RAK Medical and Health Sciences University, Ras Al Khaimah 11172, United Arab Emirates; ajaykamath@rakmhsu.ac.ae; 8 Saqr Hospital, Ministry of Health and Prevention, P.O. Box 5450, Ras Al Khaimah 72603, United Arab Emirates

**Keywords:** inflection count metric, tortuosity, morphological changes, fundus images, arteriovenous length ratio

## Abstract

Retinal imaging is a non-invasive technique used to scan the back of the eye, enabling the extraction of potential biomarkers like the artery and vein ratio (AVR). This ratio is known for its association with various diseases, such as hypertensive retinopathy (HR) or diabetic retinopathy, and is crucial in assessing retinal health. HR refers to the morphological changes in retinal vessels caused by persistent high blood pressure. Timely identification of these alterations is crucial for preventing blindness and reducing the risk of stroke-related fatalities. The main objective of this paper is to propose a new method for assessing one of the morphological changes in the fundus through morphometric analysis of retinal images. The proposed method in this paper introduces a novel approach called the arteriovenous length ratio (AVLR), which has not been utilized in previous studies. Unlike commonly used measures such as the arteriovenous width ratio or tortuosity, AVLR focuses on assessing the relative length of arteries and veins in the retinal vasculature. The initial step involves segmenting the retinal blood vessels and distinguishing between arteries and veins; AVLR is calculated based on artery and vein caliber measurements for both eyes. Nine equations are used, and the length of both arteries and veins is measured in the region of interest (ROI) covering the optic disc for each eye. Using the AV-Classification dataset, the efficiency of the iterative AVLR assessment is evalutaed. The results show that the proposed approach performs better than the existing methods. By introducing AVLR as a diagnostic feature, this paper contributes to advancing retinal imaging analysis. It provides a valuable tool for the timely diagnosis of HR and other eye-related conditions and represents a novel diagnostic-feature-based method that can be integrated to serve as a clinical decision support system.

## 1. Introduction

Various morphological abnormalities in the retinal vasculature, such as increased artery tortuosity, altered arteriovenous width and ratio (AVR), arteriovenous (AV) nicking, and the onset of macular edema in severe cases, characterize hypertensive retinopathy (HR) [1] and diabetic retinopathy [2]. These abnormalities can lead to reduced visual acuity and an increased risk of stroke later in life, suggesting inflammation in the retinal vasculature. To avoid issues like stroke, visual loss, and cardiovascular disease, it is essential to diagnose and treat HR promptly [1,3,4].

Current ophthalmology practices involve using fundus cameras and computerized images to analyze morphometric changes in retinal blood vessels for identifying and grading HR. However, early HR symptoms can be subtle and challenging to detect, leading to delays in diagnosis and complicating treatment [1,3,4]. Therefore, encouraging hypertensive patients to undergo regular follow-ups with ophthalmologists is crucial to halt the progression of retinopathy and prevent potential stroke or other severe outcomes. Developing an automated system capable of evaluating morphometric alterations in blood vessels for HR diagnosis is essential to aid in efficient treatment.

This paper introduces a novel approach called the arteriovenous length ratio (AVLR), which has not been utilized in previous studies, to address the morphological abnormalities caused by hypertensive retinopathy (HR) in the retinal vasculature or which could also be used to observe the morphological changes caused by diabetic retinopathy. Unlike commonly used measures such as the arteriovenous width ratio or tortuosity, AVLR focuses on assessing the relative length of arteries and veins. By harnessing deep learning techniques, this innovative method accurately quantifies the proposed AVLR, providing a unique perspective on the morphological changes associated with HR. Additionally, the proposed approach incorporates the analysis of vascular tortuosity and utilizes a combination of nine length feature measurements. The algorithm is trained on the AV-Classification dataset [5], consisting of 504 retinal images meticulously annotated at the pixel level for vascular segmentation, artery/vein classification, and optic disc localization. The dataset also includes image-level labels provided by ophthalmologists for vascular tortuosity indices and HR grading. This automated approach to measuring this feature holds great promise in efficiently diagnosing and grading HR, enabling timely treatment and improving patient outcomes.

The main contributions of this research work are listed below:

1. We propose a unique automated method that combines vascular tortuosity assessment with AVLR analysis, which could be used to detect and grade HR. Since this is a novel approach and is not present in existing medical literature, our study represents a substantial development in the area.
2. We provide a novel iterative approach to compute the AVLR inside the ROI (region of interest) around the optic disc using vessel caliber data. This technique improves the AVLR assessment’s precision and dependability.
3. In the assessment of vessel AVLR, we employ a unique combination of nine features, which helps with accurately quantifying the severity of AVLR and effectively minimizing the subjectivity and variability associated with manual assessments conducted by ophthalmologists.
4. The AV-Classification dataset has been expanded with new labels for AVLR metrics, creating the AV-Classification dataset extension. This extension includes vessel segments and image-level AVLR metrics, quantified using the proposed methodology.

The rest of this paper is organized as follows: Section 2 elaborates on the fundus image processing steps related to this research. Then, Section 3 presents the used material, performance measures, and the proposed method. The results, the newly prepared dataset, and the extension are explained in Section 4.7, followed by discussions in Section 4.5.4 and conclusions in Section 5.

## 2. Related Work

The evaluation of retinal vascular morphology has been studied as a means to quantify biomarkers for ocular diseases. Two key quantitative measures that have been investigated are the arteriovenous width ratio (AVR) and the arteriovenous tortuosity index. Accurately measuring these metrics requires robust vessel segmentation and classification [6].

For AVR analysis, several semi-automated approaches relying on manual input have been explored. Akbar et al. calculated AVR from manually labeled vessels [7], demonstrating its potential clinical utility. Similarly, proprietary software for semi-automated arteriolar and venular caliber measurements was used by Akbar et al. to compute AVR. However, the manual component makes these methods time-consuming, subjective, and difficult to reproduce.

To enable high-throughput screening, fully automated vessel segmentation and classification techniques are needed. Narasimhan et al. proposed a method using median filtering, top hat transform, and ROI detection to calculate AVR. However, performance was only demonstrated in a small dataset of 75 images without disease grading, thereby limiting clinical validation. The authors of [8] developed an automated technique using multi-scale linear structure enhancement and thresholding for vessel segmentation. However, AVR was only calculated within a limited region of 0.5–1 optic disc diameter around the optic disc, which may bias results and not capture overall vascular morphology. Researchers in [9] classified vessels and calculated AVR using Gabor wavelets and multilayered thresholding but did not describe the AVR measurement methodology sufficiently enough for it to be evaluated and reproduced.

More recently, advances in machine learning have enabled more reliable automated vessel analysis. Badawi et al. optimized vessel segmentation and artery/vein classification by developing B-COSFIRE filters and training convolutional neural networks (CNNs). This enabled accurate pixel-wise vessel labeling to derive vascular morphology metrics. Similarly, their proposed automated pipeline using unsupervised learning for vessel classification and wavelet transforms for diameter measurement to quantify AVR demonstrated accurate AVR measurements compared to manual grading. Further research on interpretable and robust machine learning techniques is critical in order to employ automated retinal vascular analysis for clinical use.

Additionally, Badawi et al. developed a method to automatically grade the tortuosity severity of retinal images into four levels: normal, mild, moderate, and severe. They calculated 14 tortuosity metrics for each vessel segment and used these metrics to train machine learning models to classify images. The best model, distributed random forest (DRF), achieved 99.4% accuracy on the AV-Classification dataset. This demonstrates the feasibility of automatic tortuosity severity grading.

Beyond AVR, quantifying vessel tortuosity could provide complementary information about vascular health. However, fewer studies have investigated automated measurement of the arteriovenous tortuosity index. The authors of [10] segmented vessels using k-means clustering and measured tortuosity based on the arc-chord ratio and stationary points along vessel centerlines. While promising, the methodology was only validated in two public datasets, necessitating wider evaluation. Kanski et al. proposed an index combining local and global tortuosity metrics for optical coherence tomography (OCT) images. However, only eleven OCT volumes were analyzed, limiting clinical significance.

A major limitation of the existing techniques is that most of them focus exclusively on either AVR or tortuosity, whereas simultaneously quantifying both could improve disease detection. Towards this goal, the authors of [11] presented a multi-task CNN to measure AVR and the tortuosity index end-to-end from fundus images jointly. However, its performance did not exceed single-task models, indicating that further architectural refinements are needed to learn complementary feature representations effectively.

Incorporating domain knowledge to derive interpretable biomarkers is an active area of research. Poplin et al. developed a deep learning system to predict cardiovascular risk factors by extracting interpretable vascular features, mimicking how clinicians qualitatively assess retinal images. However, the model was trained on only two datasets, requiring broad validation. Developing and rigorously evaluating hybrid machine learning systems that integrate vascular domain knowledge could advance clinical translation and adoption [12].

Emerging imaging modalities also provide new opportunities for vascular analysis. OCT angiography enables depth-resolved visualization of the retinal capillary network. However, few automated algorithms have been developed to quantify morphological changes in these volumetric scans. A key challenge is accurately segmenting the capillaries, which have low contrast and discontinuity compared to major vessels in enface projections [13].

In summary, the robust quantification of retinal vascular morphology shows strong promise as a non-invasive biomarker for systemic and ocular diseases. While progress has been made, especially in AVR measurement, substantial opportunities exist to improve interpretability, incorporate multimodal data, and move these technologies toward clinical integration. Key priorities for further research include: (1) developing unified algorithms that simultaneously quantify multiple morphology measures; (2) advancing machine learning techniques that integrate anatomical domain knowledge to derive explanatory biomarkers; (3) expanding rigorous validation across diverse patient populations and eye diseases; and (4) innovating volumetric analysis methods for emerging modalities like OCT angiography. Advances in these areas could enable more accurate disease risk stratification and unlock the full potential of retinal vascular imaging as a precision medicine tool.

## 3. Methodologies and Materials

### 3.1. Used Materials

**Figure 1:**
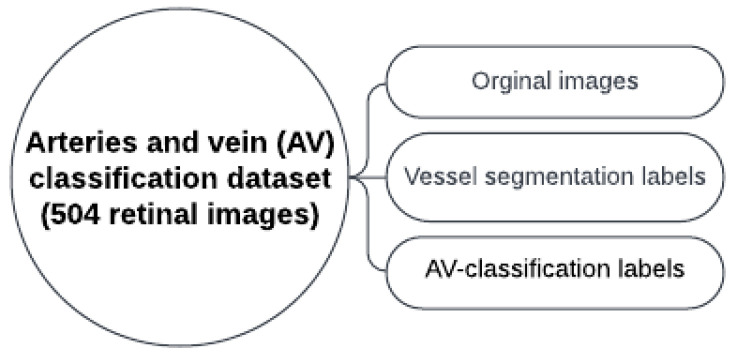
The dataset for AV-Classification utilized in this paper.

The evaluation of AVLR has undergone rigorous examination using the AV-Classification dataset [5] (See Figure 1). This comprehensive dataset comprises 504 retinal images meticulously annotated at the pixel level for vessel segmentation, artery/vein classification, and optic disc localization. The meticulous annotations were carried out by highly skilled ophthalmologists. Of the 504 images, 22 were from healthy subjects while 482 were from subjects with various forms of retinopathy, with a majority of the pathological images showing signs of hypertensive retinopathy. Other retinal vascular pathologies, like diabetic retinopathy, were also included in this dataset. All 504 images were acquired, using the same model of non-mydriatic fundus camera (Topcon), from 50 patients in the middle-aged category, ensuring consistency in image capture settings like field-of-view. There are 504 labels in total for each label type (vessel segmentation label, AV-classification label). The original retinal image from the AV-Classification dataset is fed to a deep learning optimized method to enable vessel segmentation and classification using the colored vessel segmentation labels. The type-1 and type-2 pictures and labels are 2002 by 2000 pixels in size. Each original retinal picture used in vascular segmentation and AV classification investigations has two labels: colored and monochromatic.

### 3.2. Methodology

**Figure 2:**
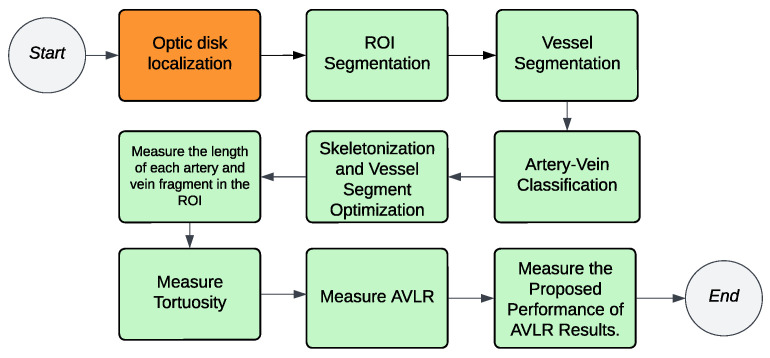
The process of measuring the proposed AVLR metric (green denotes fully automated, and orange denotes the semi-automated steps).

**Figure 3:**
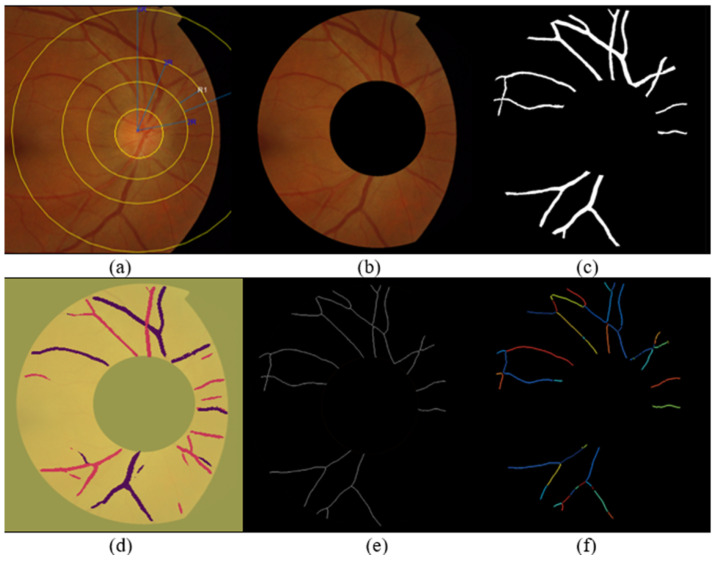
Illustration of the proposed AVLR calculation steps: (**a**) a representation of the fundus image where the optic disc is localized, (**b**) ROI segmentation, (**c**) extraction of blood vessels, (**d**) artery–vein classification (**e**) conversion to a skeletonized form, (**f**) segmentation of vessel segments into fragments where the computation for the nine metrics happens for each vessel segment; all those figures are the input to compute the AVLR for each retinal image.

The methodology for calculating the AVLR involves the application of nine metrics. Figure 2 depicts a summary of the procedure, where the workflow steps are displayed. The initial trial took place in the ROI-1 zone, specifically focusing on an area ranging from twice the size of the optic disc radius to thrice its size. The subsequent trial occurred in the R2 region, targeting a region spanning from twice the radius of the optic disc to five times its radius. The segmented and skeletonized retinal images were initially created, as depicted in Figure 3, to prepare them for further analysis. Once the segmentation was completed, the images were divided into segments. Next, every segment was subjected to the nine length metrics. These measures were created particularly to measure the length properties of the retinal arteries and veins. The AVLR may be estimated by measuring the lengths of these vessels, offering important insights into the morphological alterations connected to hypertensive retinopathy (HR) and other eye-related disorders. A thorough review procedure was carried out to verify the precision of the AVLR readings. This entailed comparing the estimated AVLR values with expert assessments and ground truth annotations. The evaluation was carried out on the AV-Classification dataset, which comprises retinal pictures that ophthalmologists have carefully tagged and categorized.

Firstly, branch points were detected using a morphological operation to identify the locations where the vessel tree branches [14]. This step is crucial for measuring the AVLR. It enables the analysis and quantification of AVLR by segmenting the detected branch points into different vessel segments. After that, another morphological process that removes the inner pixels while keeping the pixels that indicate the vessel structure was used to acquire the vessel tree’s vasculature edges [14]. Following this, vessel skeletonization was performed using an optimized vessel fragments extraction technique, outlined in [15]. This method improves the skeletonization results by progressively eliminating spur dots and smoothing the skeleton. The vascular tree generates eleven vessel branch segments after an innovative approach is used to remove bogus “L”-shaped (junction) segment sections. For the best accuracy, we used the optimal technique for extracting vessel fragments as outlined in [15]. This approach optimizes vessel segments for segment and image-wise morphometric analysis, allowing us to proceed with AVLR evaluation. The proposed methodology focuses on measuring the AVLR. It can be utilized in conjunction with other methods for comprehensive analysis, like tortuosity or arteriovenous width ratio, enabling a thorough assessment of retinal vasculature morphology. In addition, the AVLR is compared for both eyes to identify any potential differences. By examining the AVLR values from the left and right eyes, the methodology aims to detect inter-eye variations that may provide valuable insights into the asymmetry or bilateral involvement of retinal vascular changes. This comparative analysis of AVLR between the eyes adds an important dimension to the assessment, enabling a comprehensive evaluation of the retinal vasculature in relation to hypertensive retinopathy (HR) and other eye-related conditions. The table presented below provides a concise overview of the methodology employed in the paper, outlining the key steps involved in the process:

1. Optic Desk Localization: The fundus image undergoes a process to detect the location of the optic disk.
2. ROI Segmentation: The fundus images are subjected to ROI segmentation to detect and extract the ROI-2-3 ROD and 2-5 ROD from the rest of the image.
3. Vessel segmentation: The retinal images are subjected to a vessel segmentation process to separate the blood vessels from the background.
4. Artery–vein classification: The segmented vessels are analyzed to differentiate between arteries and veins in the vascular structure of the ROI.
5. Vessel skeletonization: The segmented vessels are further processed using skeletonization techniques to obtain a thin representation of the vascular structure.
6. Identification of the bifurcation/intersection points: Key points along the skeletonized vessels are identified to extract the vessel segments between them.
7. Generate the optimized vessel segments: Optimized vessel segments are generated based on the verified points, focusing on specific regions of interest.
8. Calculate each of the nine metrics long feature vectors: The nine length/tortuosity metrics are calculated for each optimized vessel segment, providing quantitative measurements for AVLR assessment.
9. Finalize the new AVLR feature set: The calculated AVLR values from each vessel segment are compiled to form a new feature set specifically for AVLR analysis.

### 3.3. Artery–Vein Classification

A deep learning strategy is employed using the author’s method in [16] to categorize retinal blood vessels as either arteries or veins. Specifically, an encoder-decoder fully convolutional neural network (FCN) architecture is utilized for this purpose.

The encoder part of the FCN is responsible for acquiring high-level distinctive features from the input retinal images. It comprises several convolutional layers, each followed by a ReLU (Rectified Linear Activation) function to introduce nonlinearity. Additionally, max-pooling layers are inserted to gradually reduce spatial dimensions and encode the most salient features.

The decoder component of the FCN operates on the encoded feature representation and generates dense pixel-wise predictions to produce a segmentation mask output. It incorporates upsampling layers to restore the original input dimensions, ensuring the output segmentation masks match the input image size. Convolutional layers and ReLU activations are also employed in the decoder.

Unlike typical convolutional neural networks, this FCN includes only convolutional layers in both the encoder and decoder, omitting any fully connected layers. This design allows it to accept input images of varying dimensions and produce corresponding output segmentation masks.

The network is trained end-to-end, utilizing the raw retinal images as input without any preprocessing steps like vessel segmentation. A multi-loss function is developed to optimize the learning of arterial and venous labels at the pixel level. This function incorporates both pixel-wise and segment-wise loss components.

The pixel-wise loss assesses predictions and ground truth labels individually for each pixel. On the other hand, the segment-wise loss introduces contextual information by considering the predominant label within each vessel segment. It adapts pixel-level predictions based on segment-wise vascular labeling. This combined approach enhances performance compared to relying solely on pixel-wise loss.

By directly learning highly distinctive features from the retinal images, the FCN can accurately classify individual pixels as artery, vein, or background, eliminating the need for hand-crafted features or pre-segmented vessel maps. The inclusion of a multi-loss function with both pixel and segment-level components further elevates the performance of artery/vein classification.

### 3.4. Vasculature Segmentation and Skeletonization

The segmentation of blood vessels in retinal images is a complex task due to various challenges, such as poor contrast, uneven lighting, and background artifacts. Despite these difficulties, several supervised and unsupervised methods have been developed for retinal vessel segmentation. In this study, we optimize the parameters of a filter to effectively segment retinal vessels based on the work in [5]. Our proposed technique uses multi-objective optimization to enhance the results obtained from the B-COSFIRE algorithm used in [17] to address issues like the central light reflex. The method successfully segments the vasculature, including the vessels, with the center light reflex. Artery–vein segmentation results are also provided using a method previously proposed in [15]. Furthermore, a thinning process removes the vascular skeleton, eliminating noise and refining the skeleton’s shape. The process involves iterative detection and elimination of endpoints, followed by removing extra pixels that create L-shaped angles and fragment the vessels.

### 3.5. Optimized Retinal Images

To generate the optimized retinal image, we utilized a blood vessel segmentation method that builds on the trainable B-COSFIRE filter. To find additional optimum parameters, the selection of the thresholding parameter was thoroughly analyzed, and background artifact removal methods were used, according to the process description in [17]. The findings from the suggested strategy outperformed those from other cutting-edge techniques used in vessel segmentation. We employed the ANOVA analysis with a *p*-value threshold of less than 0.05 to determine the significance of the parameters influencing the performance. This analysis allowed us to identify the most influential parameters in the process.

### 3.6. AVLR Metrics

**Figure 4:**
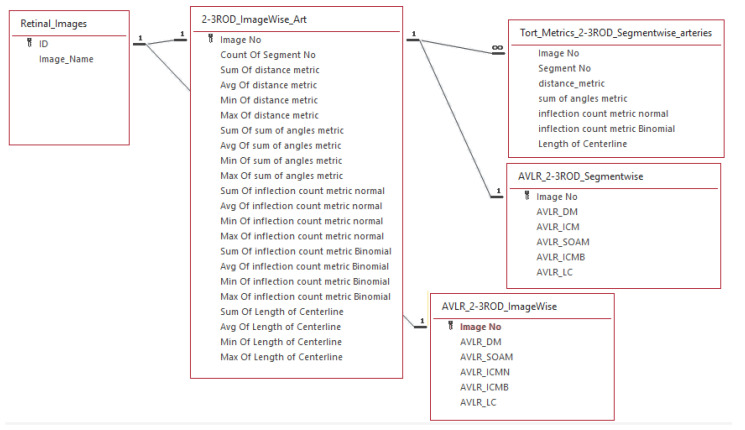
ERD diagram of the image-level and segment-level feature sets.

The process begins by traversing the extracted skeleton segments of each image. The feature extraction procedure begins by calculating the straight-line and geodesic distances inside each vascular segment. Subsequently, all nine measures of AVLR that are given in Equations (1)–(9) are calculated. This generates a record for the segment within a dedicated feature set designed to capture the length attributes of each vessel segment. In addition, the tortuosity calculation is incorporated into our metrics to capture the degree of curvature or winding in blood vessels. The structural characteristics of arteries and veins are quantitatively assessed by including tortuosity measurements, providing insights into vessel elongation, branching, kinking, and other abnormalities associated with retinal vascular diseases and systemic conditions. This integration enhances the accuracy and comprehensiveness of our assessment of vessel length attributes, enabling the derivation of the AVLR for evaluating the asymmetry and differences between arteries and veins. To provide a comprehensive overview, statistical summaries are then computed for the segments within each image, forming a row in the feature set file that captures image-level attributes. Sample metrics showcasing distance-based measures for vessel length evaluation are depicted in Figure 4, leading to the final calculation of AVLR. Below is a comprehensive description and definition of all nine length metrics along with their corresponding attributes, which are essential for the calculation of the AVLR.

1. **Arteriovenous Chord Length Ratio:** A ratio of the average Euclidean length of all veins to all arteries in the retinal picture is represented by this measure. It measures the proportional variations in length between veins and arteries.
  (1) $$Arterivenous\_SLD\_Length\_Ratio=\frac{average(Artery\_SDLE\_Length)}{average(Vein\_Chord\_Length)}.$$
2. **Arteriovenous Arc Length Ratio:** The ratio of all the arteries’ mean geodesic distance to all the veins’ mean geodesic distance in the retinal picture is represented by this measure. It sheds light on the differences and curving routes of veins and arteries.
  (2) $$Arterivenous\_Arc\_Length\_Ratio=\frac{average(Artery\_Arc\_Length)}{average(Vein\_Arc\_Length)}.$$
3. **Arteriovenous Distance Metric Ratio:** The ratio of the average tortuosity length metric of all veins to all arteries in the retinal picture is represented by this measure. The overall tortuosity and variations in vein and artery curvature are quantified.
  (3) $$Arterivenous\_DM\_Length\_Ratio=\frac{average(Artery\_DM\_Length)}{.}average(Vein\_DM\_Length)$$
4. **Arteriovenous Inflection Count Metric Ratio:** This measure shows the proportion of the average tortuosity. The total inflections measurement of each artery to the average tortuosity total inflections measurement of every vein in the retinal picture. The quantity of inflection points is counted, which represents the degree of vascular tortuosity.
  (4) $$Arterivenous\_ICM\_Length\_Ratio=\frac{average(Artery\_ICM\_Length)}{average(Vein\_ICM\_Length)}.$$
5. **Arteriovenous Inflection Count Metric Binomial Ratio:** The mean tortuosity inflection count measure binomial of all arteries divided by the average tortuosity inflection_Count_Metric_Binomial of all veins in the retinal picture is represented by this measure. It evaluates the veins’ and arteries’ binomial distribution of inflection sites.
  (5) $$Arterivenous\_ICMb\_Length\_Ratio=\frac{average(Artery\_ICMb\_Length)}{average(Vein\_ICMb\_Length)}.$$
6. **Arteriovenous Sum of Angles Metric Ratio:** This metric is the average tortuosity inflection count divided by its ratio. Comparing the average inflection count metric of all arteries to the binomial of all veins in the retinal image; it assesses the binomial distribution of artery and vein inflection points.
  (6) $$Arterivenous\_SOAM\_Length\_Ratio=\frac{average(Artery\_SOAM\_Length)}{average(Vein\_SOAM\_Length)}.$$
7. **Arteriovenous Norm of Curvature Ratio:** This measure represents the proportion of the average curvature of all vein segments to all artery segments in the retinal picture. It gauges the vessel segments’ curvature.
  (7) $$Arterivenous\_NC\_Length\_Ratio=\frac{average(Artery\_NC\_Length)}{average(Vein\_NC\_Length)}.$$
8. **Arteriovenous of Average Curvature Ratio Standard Deviation:** This statistic calculates the difference between the average curvature of all arteries’ mean standard deviation and the average curvature of all veins’ mean standard deviation. the retinal picture. Quantification is used to the curvature variation along vessel segments.
  (8) $$AV\_SDAC\_Length\_Ratio=\frac{average(Artery\_SDAC\_Length)}{average(Vein\_SDAC\_Length)}.$$
9. **Arteriovenous of Centerline Length Ratio:** This metric represents The mean centerline length of all the arteries divided by the mean centerline length of all the veins in the retinal image. It determines the distances between the arteries’ and veins’ centerlines.
  (9) $$AV\_CL\_Length\_Ratio=\frac{average(Artery\_CL\_Length)}{average(Vein\_CL\_Length)}.$$

### 3.7. The New Feature-Set Preparation

The AVLR calculation for the entire AV-Classification dataset (504 images) is performed using the proposed method. The initial step described in Figure 2 involves dividing a binary image that contains the retinal vessels into segments using an optimized method [17]. After that, iterative thinning is applied to extract the vessel skeleton. The skeleton is then broken into fragments and optimized into vessel fragments, which connect intersections, bifurcations, or endpoints in the skeleton, as explained in [5]. Each of these vessel fragments is treated as a curve, and nine mathematical formulas (Formulas (1)–(9)) are utilized to measure the length ratios and quantify the attributes of the AVLR for each fragment. This process generates a feature set at the fragment level, consisting of the AVLR metrics. Additionally, summary statistics are computed for each image to create the image-level feature set as depicted in Figure 4. The process involves evaluating the nine-length ratio metrics and incorporating the newly labeled AVLR feature set as an extension to the AV-Classification dataset, which encompasses both image-wise and vessel segment-wise tortuosity features. Two types of AVLR features are introduced: segment-level AVLR features and image-level statistics AVLR features. The image-level statistics include the count of segments in the image and, for each length metric, statistical summaries such as average, minimum, and maximum values are calculated. Figure 4 illustrates the entity-relationship diagram (ERD), representing the feature set.

### 3.8. Right and Left Eyes Comparison

The comparison of AVLR values between the left and right eyes is the final step in our analysis, providing a comprehensive assessment of any asymmetry or significant differences. This comparison allows for a detailed evaluation of the retinal characteristics of each eye and serves as a valuable tool in identifying potential abnormalities or discrepancies.

To conduct this comparison, AVLR values are obtained separately for the left and right eyes. We apply the AVLR calculation methodology outlined in the previous steps to the retinal images of each eye, recording and compiling the AVLR values for further analysis.

Once we have obtained the AVLR values for both eyes, we conduct a thorough comparison using statistical methods and techniques to assess the level of asymmetry or significant differences between the AVLR values of the left and right eyes.

The results of this comparison provide valuable insights into retinal health and potential variations between the eyes. Clinicians and researchers carefully examine any notable discrepancies or asymmetry in AVLR values and interpret these findings. These results contribute to a deeper understanding of retinal conditions and may have implications for diagnosis, treatment, and overall eye health management.

Incorporating this step into the methodology enables a comprehensive evaluation of retinal health and asymmetry between the left and right eyes, enhancing the diagnostic process and assisting in developing personalized treatment plans tailored to the specific needs of each individual.

## 4. Results

### 4.1. Optic Disc Localization

**Figure 5:**
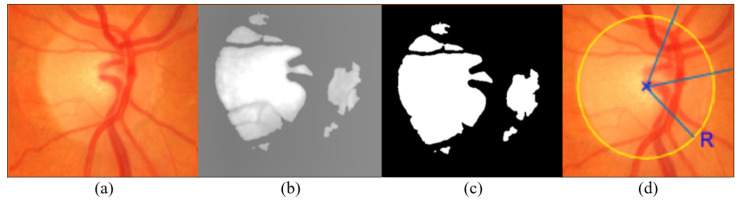
Optic disc localization illustration: (**a**) OD in the fundus image (**b**) converted to grayscale (**c**) and monochrome (**d**) the image annotated with the OD center and radius.

Figure 5 illustrates the first step in generating a ring cut image, which identifies the optic disk’s center and radius. This involves converting the image to grayscale and monochrome to isolate the circular shape. The calculated geometric center and optic disc radius serve as the basis for further image analysis and metrics.

### 4.2. ROI Segmentation

**Figure 6:**
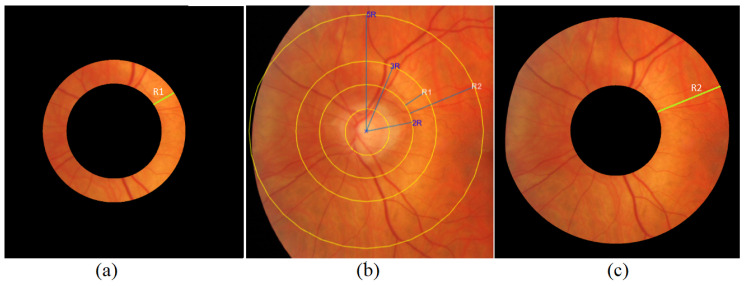
ROI segmentation: (**a**) Ring cut from 2ROD to 3ROD; (**b**) annotated original image (**c**); ring cut from 2ROD to 5ROD.

In the second stage, we create ring-cut images by segmenting the regions of interest (ROIs) with radii ranging from 2ROD to 3ROD and an extended range up to 5ROD (See Figure 6). Each segment corresponds to a new image, allowing targeted analysis of specific circular regions around the optic disk.

### 4.3. Vessel Segmentation

**Figure 7:**
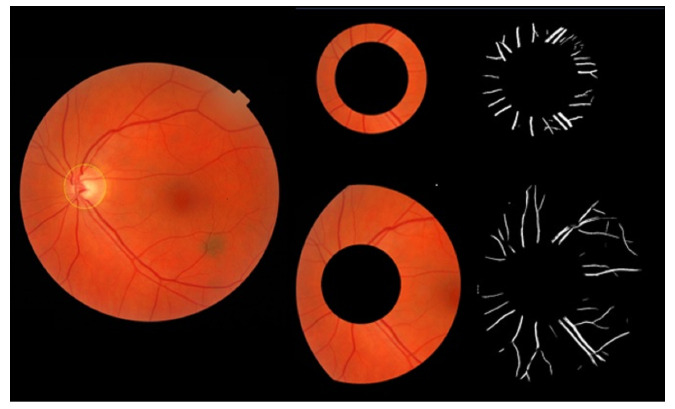
Fundus image and vessel segmentation for the two ROI areas with improved accuracy using the trainable B-COSFIRE filter and optimized parameters.

The third step utilizes the previous work of [17] to optimize blood vessel segmentation. By extending the trainable B-COSFIRE filter, more optimal parameters are identified, resulting in improved vessel segmentation accuracy; see Figure 7.

### 4.4. Artery Vein Classification

**Figure 8:**
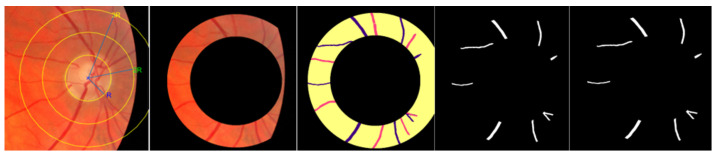
Artery–vein classification and segmentation of ROI of the segmented ring from 2ROD to 3ROD.

**Figure 9:**
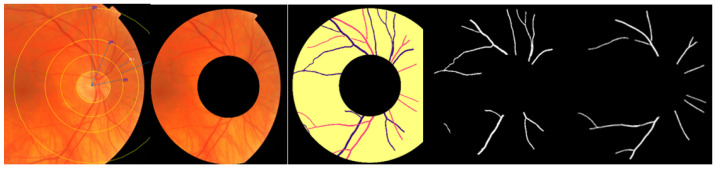
Artery–vein classification and segmentation of ROI of the segmented ring from 2ROD to 5ROD.

In the fourth step, deep learning is applied using the method in Section 3.3 to segment arteries and veins in each ring cut (Figure 8 and Figure 9). The implementation of the multi-loss technique elevates pixel accuracy from 93.5% to 97% on the dataset [16]. Following training on this extensively annotated dataset, the method attains exceptional accuracy, underscoring its efficacy in robust artery/vein classification.

#### 4.4.1. Skeletonization and Vessel Segment Optimization

**Figure 10:**
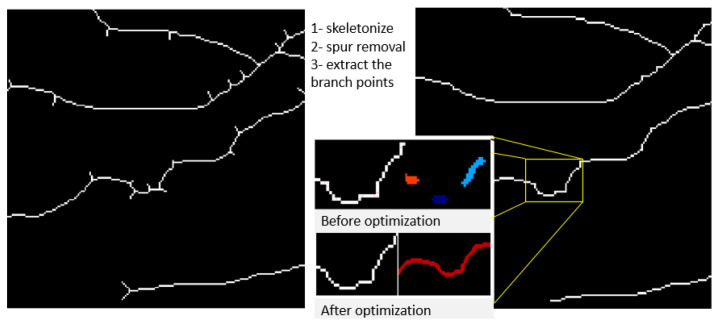
Illustration of improving the extraction of vessel segments by removing spurs after skeletonization and branch points removal.

**Figure 11:**
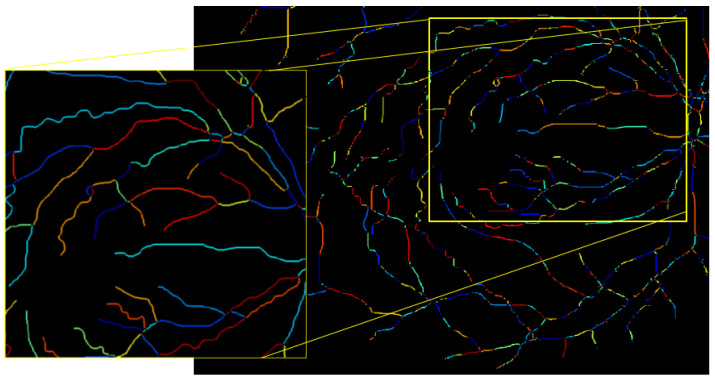
View of the enhanced vessel segments before and after the enhancement.

In the next step, the segmented vasculature is skeletonized and optimized using the method from [18], preparing the vessel segments for further processing (Figure 10 and Figure 11). These improvements result in a more accurate vessel tortuosity calculation, with significant enhancements in sigma level and confirming yield.

#### 4.4.2. Measure the Length of Each Artery and Vein Fragment in the ROI

**Figure 12:**
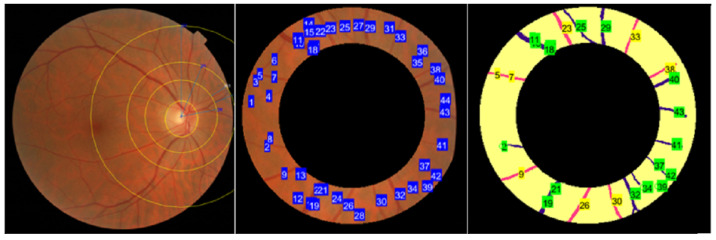
Illustration of the vessel segments that have been extracted, along with the calculation of their respective lengths.

Each extracted vessel segment is passed to calculate its length to be ready as input for tortuosity metrics calculations using the method in [5] (see Figure 12).

### 4.5. Observations on Fundus Images and Their Corresponding Metric Values

The intricate analysis of fundus images, along with their corresponding metric values, not only provide valuable insights into the complex domain of retinal morphology but also unveil important characteristics of arteriovenous length and the AVLR. In this section, we delve into a detailed discussion of selected fundus images, highlighting our observations and findings.

#### 4.5.1. Arteriovenous Length Analysis

**Figure 13:**
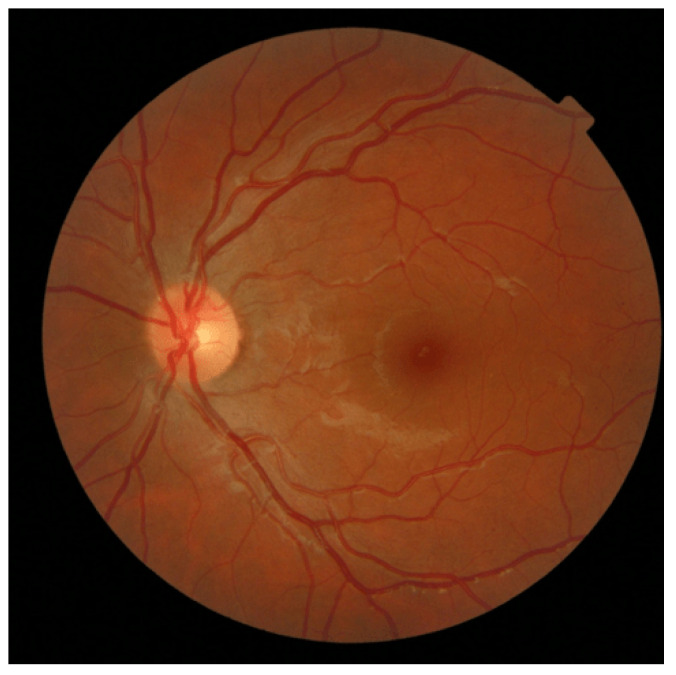
A healthy fundus image showcasing balanced vasculature with metric values: AVLR_DM (1.013), AVLR_SOAM (1.15), AVLR_ICMN (1.055), AVLR_ICMB (1.004), and AVLR_LC (0.977), signifying normalcy as the arteries’ metric values closely match the mean of the veins.

Upon a meticulous analysis of the selected fundus image metrics results, we discerned a noteworthy pattern: the reported length in the arteries was generally consistent with that of the veins, thereby reinforcing the hypothesis that the lengths of arteries and veins in the retina exhibit a considerable degree of similarity in the normal situation [18]. Figure 13 showcases a fundus image from the dataset that has this case.

#### 4.5.2. An Intriguing Observation

We made an interesting observation while curating the AV-Classification dataset, which inspired further research. We noticed that the fundus images showed clear patterns of tortuosity in the arteries and veins, which could be classified into one of three categories.

**Figure 14:**
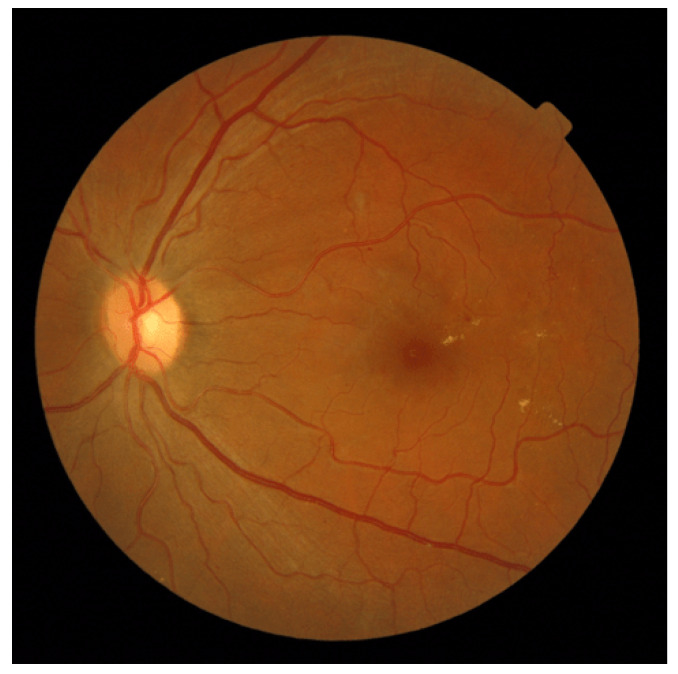
A fundus image displaying atypically elongated arteries and normal veins with metric values: AVLR_DM (7.194), AVLR_SOAM (0.843), AVLR_ICMN (29.811), AVLR_ICMB (13.785), and AVLR_LC (8.579).

**Figure 15:**
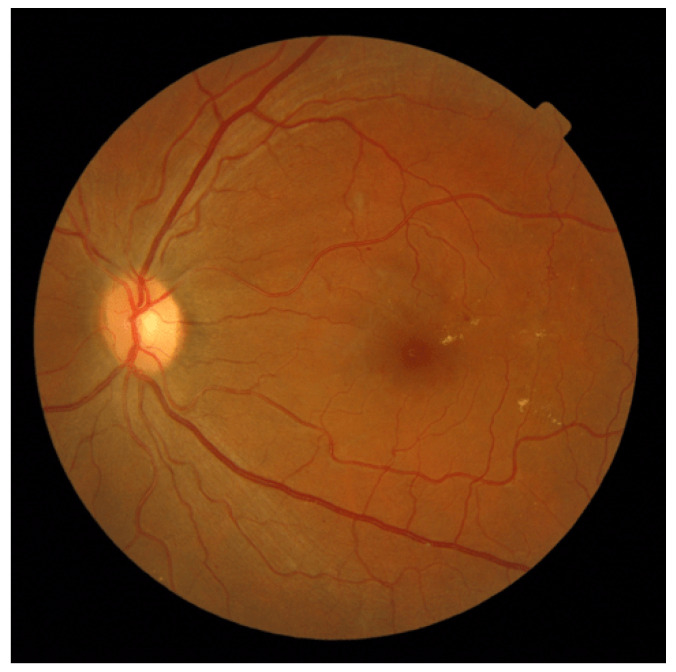
A fundus image displaying atypically elongated arteries and normal veins with metric values: AVLR_DM (0.424), AVLR_SOAM (0.942), AVLR_ICMN (0.27), AVLR_ICMB (0.615), and AVLR_LC (0.487).

- *Atypically Elongated Arteries and Normal Veins:* In a subset of retinal images within the AV-Classification dataset, we encountered instances where the arteries displayed significant elongation while the veins retained a relatively normal appearance. This divergence in vascular morphology raises intriguing questions about the potential factors driving this asymmetry and may help physicians to efficiently diagnose retinal abnormalities. Figure 14 showcases that.
- *Atypically Elongated Veins and Normal Arteries:* On the other hand, a contrasting pattern was evident in certain other fundus images. The veins showcased noticeable elongation, while the arteries preserved a normal appearance. This polarized pattern adds another layer of complexity to our understanding of retinal vascular abnormalities; such cases may be signs of specific further diagnoses.
- *Mixed Elongation:* A subset of images revealed that both the arteries and veins exhibited varying degrees of elongation or, conversely, both appeared to be normal. This variability within individual retinas underscores the intricate nature of vascular architecture and emphasizes the need for a more holistic investigation; Figure 15 showcases this.

#### 4.5.3. Asymmetry in in Eye Pairs

**Figure 16:**
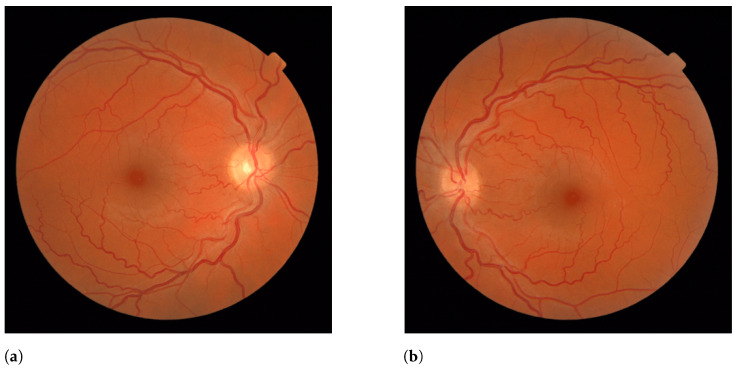
Comparison of AVLR between the right and left eyes. (**a**) Metric values for Image 1: AVLR_DM (0.102), AVLR_SOAM (1.097), AVLR_ICMN (0.042), AVLR_ICMB (0.102), AVLR_LC (0.136). (**b**) Metric values for Image 2: AVLR_DM (2.186), AVLR_SOAM (0.789), AVLR_ICMN (3.626), AVLR_ICMB (1.331), AVLR_LC (1.699).

Furthermore, our analysis led us to a specific pair of fundus images (Figure 16) that showcased a striking difference in the AVLR between the right and left eyes. The right eye displayed a severe degree of AVLR in contrast to the left eye. This pronounced asymmetry in AVLR invokes questions about its potential impact on retinal health and functionality, which warrants further investigation.

#### 4.5.4. Discussion

The myriad variations in vascular AVLR elucidate the complexities of retinal vasculature and its potential implications for ocular health. The underlying reasons for these asymmetries remain enigmatic and necessitate further exploration. Potential contributing factors could range from genetic predispositions and systemic diseases to localized pathologies impacting specific regions of the retina.

The findings put forth in this study provide pivotal insights into the diversity of retinal vascular morphologies, indicating that a generic, one-size-fits-all approach to retinal analysis may not be adequate. A comprehensive understanding of these variations could be instrumental in the early detection of ocular diseases and the development of personalized treatment strategies.

Our observations underscore the critical role of the in-depth analysis of fundus images and corresponding metric values in augmenting our understanding of ophthalmology and propelling advancements in ocular health diagnosis and care. To unravel the mysterious mechanisms governing these observed patterns and their clinical implications, further research and interdisciplinary collaboration are indispensable.

### 4.6. Observations on the Box Plot Graphs

**Figure 17:**
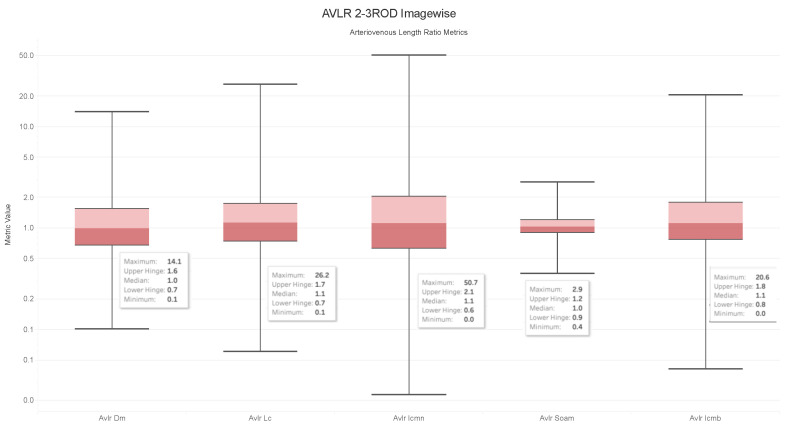
AVLR 2-3ROD Imagewise.

**Figure 18:**
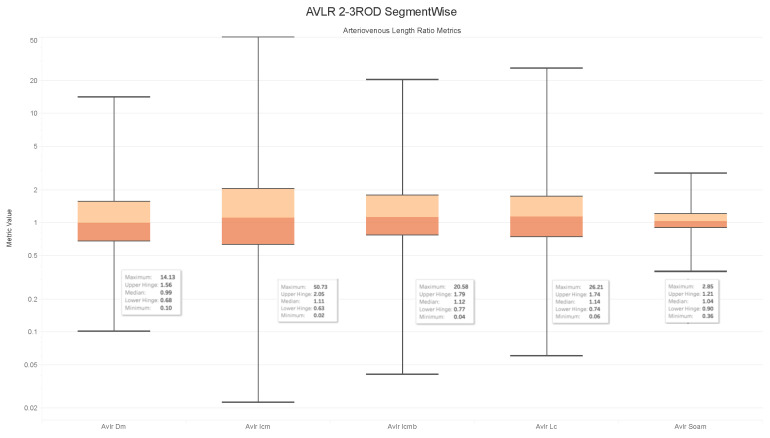
AVLR 2-3ROD Segmentwise.

**Figure 19:**
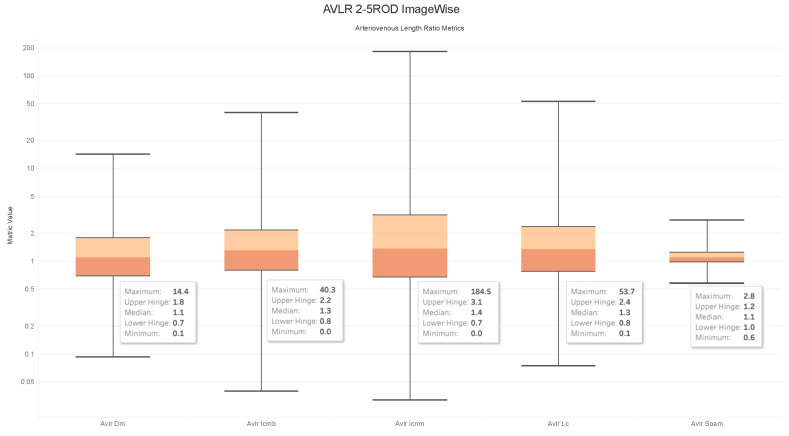
AVLR 2-5ROD Imagewise.

**Figure 20:**
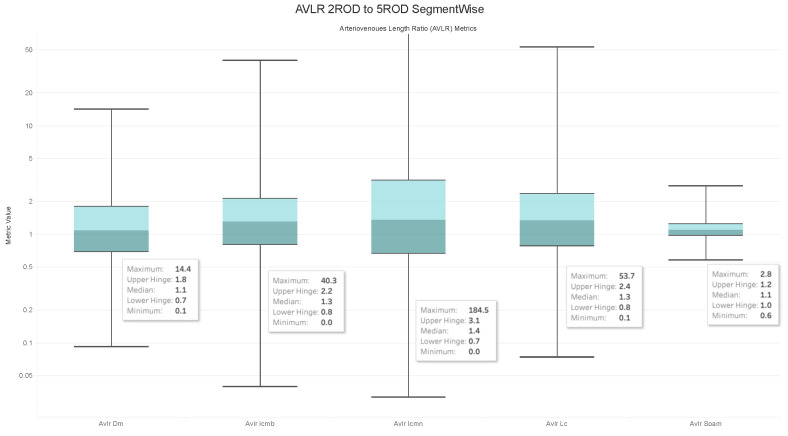
AVLR 2-5ROD Segmentwise.

Box plots of the generated AVLR metrics—specifically the ratio of the mean artery to a mean vein—for each image across the AV-Classification dataset are given in Figure 17, Figure 18, Figure 19 and Figure 20. The AVLR ratios are generated for the following metrics: inflection count metric normal (ICMN), inflection count metric binomial (ICMNB), distance metric (DM), sum of angles metric (SOAM), and length centerline (LC). The equations are explained in Section 3.6.

#### Image Level Arterioveinous Length Ratios Analysis

The above observation reaffirms our statistical findings. To delve deeper into this relationship, we compute the image-based ratios across the 504 images in the AV-Classification dataset. We calculate them for each image as given in the equations. Additionally, we compute the ratio of the average tortuosity metric of retinal arteries over the average tortuosity metric of the retinal veins for each of the following length metrics: SOAM, ICM, ICMb, DM, and LC. Figure 17, Figure 18, Figure 19 and Figure 20 show the box plots of the vessel length ratios image-wise and segment-wise using the equations in Section 3.6. Those ratios attribute each image in the AV-Classification dataset to the mean length of the arteries compared to the mean length of the veins in this specific retina. This could identify the healthiness of the vessels, whether their length varies or not, and how severe the deviation that can be noticed in each fundus image of the AV-Classification dataset is from those two ratios.

- The box plots shown in Figure 17, Figure 18, Figure 19 and Figure 20 demonstrate that, across the 504 photos in the AV-Classification dataset, all metrics have a normal distribution. This leads us to a number of findings:
- The ratios for veins and arteries often show a comparable length pattern, indicating that the morphological lengthiness of veins and arteries is almost the same, with a ratio approaching 1.
- The center value of the box plots, or the median, slightly surpasses 1, indicating that the mean length of a vein is shorter than the average length of the artery.
- The ratio moves toward the top of the box plot when the artery-length mean is higher than that of the veins. As a consequence, when the length difference grows, the retinal image point rises in the box plot to approximately 50% of the normal distribution data.
- On the other hand, the ratio veers toward the bottom of the box plot when the average length of both arteries is smaller than the average length of both veins. The retinal image point in the box plot moves below the median as the length difference decreases. This tendency is seen in around 50% of the data from the normal distribution plot.
- In order to evaluate the difference between the lengths of the arteries and veins, the arc and chord lengths may be substituted because of the normal distribution of both ratios. In the meanwhile, for certain pictures in the AV-Classification dataset, the box plot in Figure 20 shows that the arteriovenous tortuosity ratios in (4)–(9) are skewed above the mean and median. This observation leads us to the following conclusions:
- The arteriovenous SOAM ratio illustrates how the curvature angle influences the calculation and limits the findings within the constrained range of the y-axis (0 to 360) by displaying a regular distribution with a mean and median of 1. However, any possible distinctions between veins and arteries may not be readily apparent because of this narrow range.
- The lengths of the arteries and veins lengthen and the ratio becomes closer to one when they have the same degree of AVLR. While the mean and median of the other AVLR ratios range from 0.99 to 1.4, the AV SOAM ratio has both a mean and a median of 1. The variations in the artery and vein diameters and their visibility on the retinal surface in each picture may be ascribed to this deviation.
- The ratio moves to the top part of the box plot if the average length of veins is shorter than the average length of arteries. This implies that the tortuosity of arteries is much greater than that of veins in the retinal image.
- Conversely, in the event that the average length of the arteries is lower than the mean lengths of the veins, the ratio will shift towards the lower end of the box plot. Vein tortuosity does not deviate substantially from the median in the box plots shown in Figure 17, Figure 18, Figure 19 and Figure 20, suggesting that fewer cases of vein tortuosity arise in veins alone.

Based on the examination of the aforementioned arteriovenous length, it can be inferred that the observed results align with the statistically calculated outcomes, indicating that the measured lengths in the arteries are equivalent to those in the veins. However, the motivation for this study originates from a finding made during the creation of the AV-Classification dataset.

- The retinal images in the AV-Classification dataset display inconsistencies in the length and tortuosity of arteries and veins.
- Specifically, some images show tortuous arteries with normal veins, while others have tortuous veins but normal arteries.
- Additionally, there are images where both artery and vein lengths are either tortuous or normal.

Although numerous studies have focused on evaluating the tortuosity of retinal vessels, there is currently no formula available to specifically identify abnormalities in either veins, arteries or both. Such phenomena present a novel avenue for future research in ophthalmology, as it marks the first exploration of this aspect within the field. Furthermore, it is worth noting that existing tortuosity calculation methods found in the literature primarily assess overall tortuosity, whereas this study delves into the distinction in arteriovenous length behavior. The calculations for the arteriovenous length ratio (AVLR) were conducted for all segments of arteries and veins in each of the 504 retinal images from the AV-Classification dataset. Subsequently, formulae were developed to describe these phenomena, and statistical analysis demonstrated that, From a geometric standpoint, there are parallels in the tortuousness of veins and arteries. The quantification discussed by the authors is regarded as a distinctive and encouraging avenue for further exploration within the domain of retinal image pathology diagnosis.

### 4.7. AVLR New Dataset

The retinal vessel morphometry (RVM) research in [15] has been further enhanced by incorporating new labels for AVLR metrics, constituting an extension named the AV-Classification dataset. This extension encompasses vessel segment-wise and image-wise AVLR metrics quantified using the methodology proposed in this paper.

**Figure 21:**
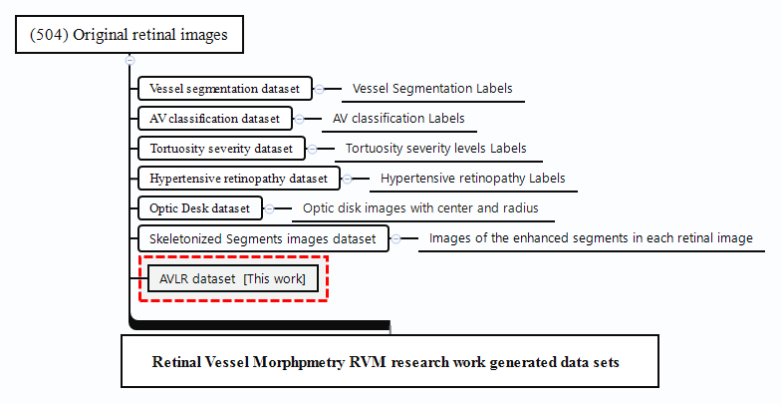
The AVLR dataset extends prior RVM work with new metrics quantified using the proposed methodology.

Specifically, as illustrated in Figure 21, the dataset now includes the following new AVLR metrics computed for each optimized vessel segment:

- Arteriovenous chord length ratio;
- Arteriovenous arc length ratio;
- Arteriovenous distance metric ratio;
- Arteriovenous inflection count metric ratio;
- Arteriovenous inflection count metric binomial ratio;
- Arteriovenous sum of angles metric ratio;
- Arteriovenous norm of curvature ratio;
- Arteriovenous standard deviation of average curvature ratio;
- Arteriovenous centerline length ratio.

Additionally, image-level statistics are calculated by aggregating the segment-wise metrics for each image. The image-level statistics consist of summary values like count, mean, standard deviation, minimum, maximum, and median for all the above AVLR metrics.

In total, the enhanced AV-Classification dataset contains over 17,000 newly labeled AVLR values spanning across vessel segments and images. This expanded dataset with the additional AVLR annotations serves as a valuable resource for research and analysis of retinal vasculature morphology. The inclusion of the new AVLR metrics significantly augments the RVM dataset, providing a more comprehensive set of labels to quantify arteriovenous asymmetries and vascular abnormalities associated with various diseases.

The AV-Classification dataset will be made publicly available to the research community upon request, enabling further advancements in this domain. The dataset can facilitate the development of machine-learning models for automated AVLR computation from retinal images. In addition, the detailed AVLR labels can help identify morphological patterns and aid in screening vascular conditions like hypertensive retinopathy. Overall, the extended dataset allows a more in-depth analysis of retinal vessel morphology and its relationship to systemic diseases.

## 5. Conclusions

In conclusion, this paper proposes a novel method for evaluating retinal vasculature morphology and its diagnostic potential in hypertensive retinopathy (HR) and other eye-related diseases. The method introduces a new approach called the AVLR, which focuses on assessing the relative length of arteries and veins in the retinal vasculature. The methodology involves segmenting the retinal blood vessels, distinguishing between arteries and veins, and measuring the length of both within the area of interest encompassing the optic disc using an iterative approach. The effectiveness of the AVLR measurement was evaluated using the RVM dataset, demonstrating superior performance compared to existing techniques.

The major contributions of this research include the development of an automated system that integrates AVLR analysis and vessel tortuosity evaluation for the detection and grading of HR. This system provides a unique perspective on the morphological changes associated with HR and offers a valuable tool for the timely diagnosis of HR and other eye-related conditions. The methodology incorporates nine metrics that can accurately quantify the severity of AVLR, minimizing subjectivity and variability with manual assessments. Additionally, the comparative analysis of AVLR between the eyes enables a comprehensive evaluation of retinal vasculature in relation to HR and other conditions.

Finally, we enhanced prior retinal vessel morphometry research by adding new labels for metrics in an extended dataset called AV-Classification. This incorporates vessel segment-wise and image-wise AVLR metrics quantified using the proposed methodology.

The proposed method holds great promise for improving the efficiency and accuracy of HR diagnosis, facilitating timely treatment, and improving patient outcomes. Further research and validation on larger datasets and in clinical settings are warranted to fully establish the diagnostic potential of the AVLR and its integration into clinical decision support systems for ophthalmology.

## Data Availability

Data are contained within article and our previous publication [16].

## Abbreviations

The following abbreviations are used in this manuscript:

HR	Hypertensive Retinopathy
AVLR	Arteriovenous Length Ratio
AVR	Arteriovenous Ratio
ROI	Region of Interest
ROD	Radius of Optic disc
AV	Arteriovenous
DM	Distance Metric
SOAM	Sum of Angles Metric
ICM	Inflection Count Metric
ICMB	Inflection Count Metric Binomial
NC	Norm of Curvature
SDAC	Standard Deviation of Average Curvature
CL	Centerline Length
FCN	Fully Convolutional Neural Network
CNN	Convolutional Neural Network
OCT	Optical Coherence Tomography
RVM	Retinal Vessel Morphometry
